# Exploring the role of combined external beam radiotherapy and targeted radioligand therapy with [^177^Lu]Lu-PSMA-617 for prostate cancer - from bench to bedside

**DOI:** 10.7150/thno.93249

**Published:** 2024-04-08

**Authors:** Daria Arbuznikova, Aikaterini Klotsotyra, Lisa Uhlmann, Lisa-Charlotte Domogalla, Nils Steinacker, Michael Mix, Gabriele Niedermann, Simon K.B. Spohn, Martin T. Freitag, Anca L. Grosu, Philipp T. Meyer, Christian Gratzke, Matthias Eder, Constantinos Zamboglou, Ann-Christin Eder

**Affiliations:** 1 Department of Nuclear Medicine, Medical Center - University of Freiburg, Faculty of Medicine, University of Freiburg, Freiburg, Germany.; 2 Division of Radiopharmaceutical Development, German Cancer Consortium (DKTK), partner site Freiburg, Freiburg, Germany and German Cancer Research Center, Heidelberg, Germany.; 3 Department of Radiation Oncology, Medical Center - University of Freiburg, Faculty of Medicine, University of Freiburg, Freiburg, Germany.; 4 German Cancer Consortium (DKTK), partner site Freiburg, a partnership between the German Cancer Research Center and Medical Center - University of Freiburg, Freiburg, Germany.; 5 Berta-Ottenstein-Program, Faculty of Medicine, University of Freiburg, Freiburg, Germany.; 6 Department of Urology, Medical Center - University of Freiburg, Faculty of Medicine, University of Freiburg, Freiburg, Germany.; 7 German Oncology Center, European University Cyprus, Limassol, Cyprus.

**Keywords:** prostate cancer, external beam radiotherapy, prostate-specific membrane antigen, targeted radioligand therapy, combination therapy

## Abstract

Management of prostate cancer (PC) might be improved by combining external beam radiotherapy (EBRT) and prostate-specific membrane antigen (PSMA)-targeted radioligand therapy (RLT) with lutetium-177 (^177^Lu)-labeled PSMA inhibitors. We hypothesized a higher efficacy of the combination due to augmentation of the radiation dose to the tumor and interactions of EBRT with PSMA expression potentially increasing radiopharmaceutical uptake. Therefore, this study analyzed the influence of radiation on PSMA expression levels *in vitro*. The results were translated to evaluate the efficacy of the combination of photon EBRT and [^177^Lu]Lu-PSMA-617 in a murine PC xenograft model. Finally, a clinical case report on a combined elective field EBRT with RLT dose escalation illustrates a proof-of-concept.

**Methods:** PSMA gene and protein expression were assessed in human PSMA-overexpressing LNCaP cells after irradiation using reverse transcription quantitative polymerase chain reaction (RT-qPCR), flow cytometry and On-Cell Western assays. In the *in vivo* therapy study, LNCaP tumor-bearing BALB/c nu/nu mice were irradiated once with 2 Gy X-ray EBRT and injected with 40 MBq [^177^Lu]Lu-PSMA-617 after 4 h or received single or no treatment (n = 10 each). Tumor-absorbed doses by [^177^Lu]Lu-PSMA-617 were calculated according to the Medical Internal Radiation Dosimetry (MIRD) formalism after deriving time-activity curves using a gamma probe. An exemplified patient case is demonstrated where fractionated EBRT (54 Gy to prostate; 45 Gy to pelvic lymphatics) and three cycles of [^177^Lu]Lu-PSMA-617 (3.4-6.0 GBq per cycle) were sequentially combined under concurrent androgen deprivation for treating locally advanced PC.

**Results:** At 4 h following irradiation with 2-8 Gy, LNCaP cells displayed a PSMA protein upregulation by around 18% relative to non-irradiated cells, and a stronger upregulation on mRNA level (up to 2.6-fold). This effect was reversed by 24 h when PSMA protein levels were downregulated by up to 22%. Mice treated with the combination therapy showed significantly improved outcomes regarding tumor control and median survival (p < 0.0001) as compared to single or no treatment. Relative to monotherapy with PSMA-RLT or EBRT, the tumor doubling time was prolonged 1.7- or 2.7-fold and the median survival was extended by 24% or 60% with the combination, respectively. Additionally, tumors treated with EBRT exhibited a 14% higher uptake of the radiopharmaceutical as evident from the calculated tumor-absorbed dose, albeit with high variability in the data. Concerning the patient case, the tri-modality treatment was well tolerated and the patient responded with a long-lasting complete biochemical remission for five years following end of PSMA-RLT. The patient then developed a biochemical relapse with oligo-recurrent disease on follow-up imaging.

**Conclusion:** The present preclinical and clinical data demonstrate that the combination of EBRT with dose escalation by PSMA-RLT improves tumor control and potentially prolongs survival. This may pave the way for further clinical investigations of this approach to explore the curative potential of the combination therapy.

## Introduction

Cancer-targeting radiopharmaceuticals represent a powerful tool in the repertoire of anticancer agents. A promising example is prostate-specific membrane antigen (PSMA)-targeted radioligand therapy (RLT) with lutetium-177 (^177^Lu)-labeled PSMA-617 ([^177^Lu]Lu-PSMA-617), which was approved by the U.S. Food and Drug Administration and European Medicines Agency in 2022 for the treatment of metastatic castration-resistant prostate cancer (mCRPC). Currently, the drug is also being investigated in earlier disease stages such as hormone-sensitive oligo-metastatic PC (NCT04443062). [^177^Lu]Lu-PSMA-617 specifically binds to PSMA which is a type II transmembrane protein **1** overexpressed in PC **2**. In response to binding of an inhibitor, PSMA undergoes internalization distributing the inhibitor throughout the cytoplasm **3**. [^177^Lu]Lu-PSMA-617 was shown to efficiently internalize **4**, allowing an accumulation of radioactivity inside of the cancer cells. The main type of radiation emitted from ^177^Lu is constituted by beta-minus particles that have a very low penetration range in tissues (mean distance of 0.67 mm) **5**. This significantly limits irradiation of healthy tissue in proximity to the tumor; however, it simultaneously makes therapy success reliant on a homogeneous PSMA expression. The beta radiation inflicts DNA damage in the form of single strand breaks (SSBs), double strand breaks (DSBs) and oxidative base lesions **6,7** which ultimately results in cell death.

Part of the standard of care in the early stage of PC is external beam radiotherapy (EBRT). An emerging approach to increase efficacy of EBRT is the combination with RLT. Two phase I and one phase II studies examining such a combination treatment are ongoing (NCT05079698; NCT05162573; NCT05560659). Results have so far only been published for a phase I trial (NCT05079698) of the administration of PSMA-RLT followed by stereotactic body radiotherapy 8. Preclinical studies on the combination therapy for PC are lacking with only one publication reporting the combined administration of proton EBRT and [^177^Lu]Lu-PSMA-617 **9**. The combination therapy might be promising for localized, locally advanced, and oligo-metastatic PC since tumors are low in number and, therefore, their treatment with EBRT is feasible, and might offer several advantages. It could allow an escalation of the radiation dose to the tumors while sparing healthy tissue. The two therapy modalities are using different routes of application and, hence, have different organs at risk. With EBRT, off-target effects are defined depending on the localization of organs relative to the radiation beam tracks and may arise in the skin, bladder and rectum, whereas with PSMA-RLT the kidneys, salivary glands and the bone marrow may experience mostly low grade toxicities 5. With less limits to dose escalation imposed by normal tissue toxicity, DNA damage can be potentiated increasing the chance of overwhelming the DNA repair capacity and inflicting lethal damage. Furthermore, addition of PSMA-RLT can eliminate micrometastases and lesions that were not detected with diagnostic imaging, thereby, improving the curative potential and possibly delaying the start of anti-hormonal therapy.

Additionally, the combination of EBRT and PSMA-RLT might also interact in a synergistic manner via the modulation of the radiopharmaceutical target expression. PSMA might play a role in the repair of DNA damage as it was shown to stimulate phosphatidylinositol-3-kinase (PI3K)/ Akt-mediated survival signaling **10** which, in turn, facilitates DSB repair **11**. PSMA was also reported to promote telomere stability in an Akt-dependent manner while inhibition of PSMA increased telomere DNA DSB formation 12. Furthermore, membranous PSMA expression was higher in DNA repair-deficient cells **13** suggesting that PSMA might compensate for reduced repair capacity in that genetic background. PSMA internalization activity upon binding of a substrate and subsequent potential for reincorporation into the membrane **3** suggest that cell surface PSMA levels can fluctuate in response to external stimuli. Therefore, after applying smaller amounts of radiation, PSMA could be upregulated to enhance DNA damage repair whereas cell death resulting in reduced PSMA density is expected after significant irradiation. Based on the biological functions of PSMA, we hypothesize that single exposure to low or clinically relevant EBRT doses (i.e., 0.5 to 8 Gy) initially upregulates PSMA levels in PC cells. Such an effect might provide a benefit for PSMA-RLT due to the potential for enhanced radiopharmaceutical uptake. Therefore, we analyzed changes in PSMA protein levels specifically on the cell surface after a single fraction of external irradiation *in vitro*. Effects on gene expression levels were also assessed. In the next step, the efficacy of the combination therapy with photon EBRT followed by PSMA-RLT using [^177^Lu]Lu-PSMA-617 was evaluated in a xenograft mouse model for PC. Finally, a patient experience with elective EBRT to the prostate and pelvic lymphatics including a dose escalation by applying PSMA-RLT is reported. We intended to reduce the EBRT dose to protect the neighboring healthy organs, and to exploit the benefit of the systemic effect of RLT.

## Materials and methods

### Cell culture and irradiation

The prostate cancer cell lines LNCaP (high PSMA-expressing, androgen-sensitive; *ATCC, CRL-1740™*) and PC-3 (PSMA-negative; *ATCC, CRL-1435™*) were cultured in Roswell Park Memorial Institute (RPMI)-1640 medium with GlutaMAX™ (*#61870010, Life Technologies™ Ltd.*) supplemented with 10% fetal bovine serum (FBS) (*#10270-106, Life Technologies GmbH*) and 1 mM sodium pyruvate (*#11360-039, Life Technologies™ Ltd.*) at 37 °C in a 5% CO_2_ atmosphere. Cell lines were regularly authenticated (last: March 2023) and monitored for mycoplasma contamination. For experiments, cells were detached with a 0.05% trypsin solution (*#P10-023100, PAN-Biotech*), counted and seeded at densities of 1.5-2x10^5^ cells/mL medium (LNCaP) or 1x10^5^ cells/mL medium (PC-3). Cells were allowed to adhere overnight after seeding. Irradiation of cells was performed using the Gammacell^®^ 40 Exactor (*Best Theratronics Ltd.*) equipped with two caesium-137 (^137^Cs) sources (maximum activity: 95 TBq). Cells were irradiated at a dose rate of 0.52-0.57 Gy/min (irradiation times: 53-56 s, 228 s, 423-445 s or 846-890 s depending on the intended dose). Control cells that were not exposed to radiation were also kept outside of the incubator for the irradiation time of the treated cells. Subsequently, the cells were placed back into the incubator for different periods of time.

### Flow cytometry

To analyze cell surface PSMA levels, flow cytometry was performed on living cells. 3x10^5^ LNCaP cells per well were cultured in 6-well plates. To collect each sample for staining, cells were detached with warm trypsin and the single-cell suspensions were then kept on ice. Cells were incubated in staining buffer (phosphate buffered saline (PBS) with 5% FBS) containing 1:167 diluted anti-PSMA antibody (6 µg/mL) that was conjugated to Alexa Fluor™ 488 (IgG1 isotype, *#ab187570, Abcam*) for 1 h on ice. Following incubation, the cells were washed three times with staining buffer and resuspended in PBS with 1 µg/mL propidium iodide (PI) for discrimination of living and dead cells. The samples were analyzed at the LSRFortessa™ Cell Analyzer (*BD Biosciences*). 1x10^4^ cells were captured per sample. After gating the main population in the forward scatter (FSC)/ side scatter plot (SSC), the Alexa Fluor™ 488 median fluorescence intensity was evaluated in living cells (**Figure S1**).

### On-Cell Western Assay (OCW)

To confirm the radiation-induced changes in surface PSMA protein levels, as previously measured by flow cytometry, an OCW experiment was carried out. The OCW is a sensitive fluorescence-based technique employing antibodies labeled with near-infrared dyes that allows the measurement of cell surface protein levels in fixed, non-permeabilized cells. The technique was adapted based on the In-Cell Western™ protocol by LI-COR, Inc. 2x10^4^ LNCaP or 1x10^4^ PC-3 cells per well were seeded in black-walled, 0.01% poly-L-lysine-coated (*#P4707, Sigma-Aldrich Co. LLC*) 96-well plates (*#3603, Corning Inc.*) in triplicate. The medium was aspirated and cells were immediately fixed in 3.7% formaldehyde (*#252549, Sigma-Aldrich Co. LLC*) in PBS for 20 min at room temperature (RT). The cells were blocked with Intercept™ Blocking Buffer (PBS) (*#927-70001, LI-COR, Inc.*) for 1 h at RT. A primary rabbit anti-PSMA antibody (*#12702, Cell Signaling Technology, Inc.*) was diluted 1:400 in blocking buffer and incubated at 4 °C overnight. The secondary antibody IRDye goat anti-rabbit 800CW (*#926-32211, LI-COR, Inc.*) was diluted 1:800 in blocking buffer and incubated at RT for 1 h protected from light. Subsequently, the cells were permeabilized using PBS with 0.2% Tween 20 (*#P1379, Sigma-Aldrich Co. LLC*) at RT to allow the incorporation of an unspecific cell dye for signal normalization in the following step. The cells were labeled with the CellTag™ 700 non-specific cell stain (*#926-41090, LI-COR, Inc.*) in PBS at a concentration of 0.2 µM for 30 min at RT and washed afterwards. Prior to scanning, all liquid was removed from the wells and the plates were immediately scanned detecting fluorescence in both 700 (cell stain) and 800 nm channels (PSMA) on the Odyssey^®^ CLx Imager (*LI-COR, Inc.*). Scans were acquired at 169 µm resolution with medium scan quality. The intensity setting applied for both channels was “Auto Mode”. The scans were evaluated using the ImageStudio™ Software (*LI-COR, Inc.*). Wells stained with the secondary antibody only were used for cell line-specific background subtraction in the 800 nm channel. Background-subtracted signal intensity values in the 800 nm channel were normalized to the signal intensity values in the 700 nm channel to account for varying cell density.

### Reverse transcription quantitative polymerase chain reaction (RT-qPCR)

The expression of PSMA on mRNA level (*FOLH1*) following ionizing irradiation was analyzed by dye-based RT-qPCR. *FOLH1* expression levels were normalized to *GAPDH* which was reported to be a suitable reference gene in radiation research **14**. 1x10^6^ LNCaP cells were seeded in 6 cm dishes. They were irradiated on the following day and incubated for 4 h after the treatment. At the end of the incubation time, total RNA was isolated using the High Pure RNA Isolation Kit (*#11828665001, Roche Diagnostics GmbH*) and RNA purity was verified using the DS-11+ spectrophotometer (*DeNovix Inc.*) by measuring the absorbance ratio at 260/280 nm. Complementary DNA (cDNA) was reverse-transcribed from 1 µg total input RNA and with gene-specific reverse primers at a final concentration of 0.1 µM using the SuperScript™ IV First-Strand Synthesis System (*#18091050, Thermo Fisher Scientific Inc.*). A control reaction without reverse transcriptase was included to rule out that presence of residual genomic DNA influences PCR results. After reverse transcription, cDNA samples were stored at -20 °C. The cDNA was purified with the NucleoSpin^®^ Gel and PCR Clean-up kit (*#74060950, Macherey-Nagel GmbH & Co. KG*) and its concentration and purity were determined using the DS-11+ spectrophotometer. qPCR was then performed using the PowerTrack™ SYBR™ Green Master Mix (*#A46012, Thermo Fisher Scientific Inc.*) with 5 ng cDNA per sample in 10 µL reactions. Each primer was added at 400 nM final concentration. Reactions were prepared in triplicate. The qPCR was run on the LightCycler^®^ 480 (*Roche Diagnostics GmbH*) and the thermal program was defined as follows: The enzyme was activated at 95 °C for 2 min. The denaturation step was carried out at 95 °C for 15 s, and primer annealing and DNA extension at 60 °C for 60 s in one step. The denaturation, annealing and extension steps were repeated for 40 cycles. SYBR™ Green fluorescence was measured at the end of each extension. After the last cycle, a melting curve analysis was performed to verify the specificity of the amplification. Details on the primers are provided in **Table S1**. Primer sequences for *FOLH1* (sequence accession number: NM_004476.3) and *GAPDH* (sequence accession number: NM_002046) gene expression analysis were identified in the PrimerBank database (*Center for Computational and Integrative Biology, Massachusetts General Hospital, Boston, MA, USA*) and ordered from Eurofins Genomics Germany GmbH. The PrimerBank IDs are 4758398a1 and 378404907c2, respectively. Primer specificity was verified using Primer-BLAST (*National Center for Biotechnology Information (NCBI), National Library of Medicine, National Institutes of Health, Bethesda, MD, USA*). The fold gene expression of *FOLH1* normalized to *GAPDH* in irradiated cells compared to non-irradiated cells was calculated using the ∆∆Ct method **15**.

### Radiolabeling

PSMA-617 was synthesized and purified in-house as published previously **4**. No-carrier-added, radiochemical grade [^177^Lu]LuCl_3_ in 0.04 M HCl was obtained from ITM Medical Isotopes GmbH or Isotopia Molecular Imaging Ltd. For radiolabeling, 58 µL of a 2.1 M 2-[4-(2-hydroxyethyl)piperazin-1-yl]ethanesulfonic acid (HEPES) (*#9105.2, Carl Roth GmbH & Co. KG*) buffer at pH 7 and 12 µL [^177^Lu]LuCl_3_ (activity: 280 ±10 MBq) were added to 2.8 nmol PSMA-617 in dimethyl sulfoxide (*#D8418, Sigma-Aldrich Co. LLC*) (specific activity 100 MBq/nmol). The final pH was adjusted to 7. The mixture was heated at 95 °C for 30 min. The radiochemical yield was determined by reversed-phase high performance liquid chromatography (HPLC) and thin-layer chromatography (TLC) using a mobile phase composed of 1:1 acetonitrile and water. Radiochemical yields obtained were at least > 98% and > 96% according to HPLC and TLC, respectively.

### *In vivo* therapy study

Male, six- to seven-week old BALB/c *nu/nu* mice (*Janvier Labs*) were subcutaneously inoculated with 1x10^7^ LNCaP cells in 50% matrigel (*#356234, Corning Inc.*) in the right flank under 2% isoflurane anesthesia (*Piramal Critical Care B.V.*). The tumors were allowed to grow to a size of ~7-9 mm in length. Four treatment groups were defined: “Untreated”, “EBRT only”, “PSMA-RLT only” and “combination”. Starting conditions on the day of treatment were similar between the four groups, as there were no significant differences in mean tumor size at this time point (one-way ANOVA, p > 0.05; **Figure S2**). EBRT was delivered by the Radsource RS2000 irradiator (*Rad Source Technologies, Inc.*) generating x-rays with an energy of 160 kV. X-rays were passed through a 0.3 mm copper filter and the mice were shielded with lead exposing only the tumors. Before irradiation, mice were sedated by intraperitoneal injection of ketamine (80 mg/kg body weight; *Medistar Arzneimittelvertrieb GmbH*) and medetomidine (0.8 mg/kg body weight; *Orion Corporation*). The tumors were irradiated with 2 Gy at a dose rate of 3.74 Gy/min. Animals from the PSMA-RLT or combination groups were anesthetized with 2% isoflurane and injected with ~40 MBq [^177^Lu]Lu-PSMA-617 in 0.9% saline (400 pmol) via tail vein. The selected RLT dose fits into the lower half of a range of published doses applied in preclinical studies on mice bearing PC xenografts **16,17,18**. The untreated and EBRT only groups received vehicle injections of 0.9% saline instead of PSMA-RLT. Animals that received combination therapy were first irradiated and administered with [^177^Lu]Lu-PSMA-617 4-5 h after irradiation. Both EBRT or/and PSMA-RLT were given once per respective group (except untreated group). On the day of treatment and then three times per week, the tumor size was measured with a caliper and the body weight was recorded. Tumor volumes were calculated using the formula length×width×height/2. Survival was evaluated according to Kaplan-Meier. Survival time was defined as the time until a humane endpoint was reached with the day of treatment considered as day 1. Defined endpoints were for example reaching the tumor size limit of 15 mm in length or ulceration of the tumor. All animal experiments were approved by the regional authority Regierungspräsidium Freiburg (approval number G18/73) and were carried out in compliance with the current laws of the Federal Republic of Germany and German Animal Welfare guidelines.

### Tumor dosimetry

A calibrated gamma probe (Crystal Probe -automatic- (SG04), *Crystal Photonics GmbH)* was utilized to quantify the background corrected activity of the tumor for about two weeks (each day or every second or third day) after injection of [^177^Lu]Lu-PSMA-617. The resulting time-activity curves of the tumor were modeled using a monoexponential fit to calculate the time-integrated activity (TIA). Tumor size (length, width, height) was measured three times using a caliper (at the start, middle and end of the gamma probe measurement period). Skin thickness measurements were obtained with a caliper from some sacrificed animals and these values were subsequently subtracted twice from the tumor size for each variable. Tumors were treated as ellipsoids when calculating their volume. The absorbed dose was calculated according to the Medical Internal Radiation Dosimetry (MIRD) formalism **19** using TIA and S values for spherical tumors **20** as an approximation for ellipsoidal tumors with the same volume. The absorbed fraction of the full lutetium-177 beta spectrum was used to further correct the S values for changes of tumor volume during the period of measurements **21**.

### Statistical analysis

Statistical hypothesis testing was performed in GraphPad Prism (version 10.0.0, *GraphPad Software, Inc*.). For the cell experiments, changes in PSMA expression levels in irradiated cells were tested for significance relative to non-irradiated controls. Flow cytometry data (median fluorescence intensity values) was normalized to control cells and evaluated by the non-parametric Kruskal-Wallis test and Dunn's multiple comparisons test. Fold gene expression values and the cell density- and control-normalized data obtained with OCW were analyzed for significance using the same tests. For the therapy study, mean differences in the course of tumor growth were assessed between all possible group combinations by the Mixed-Effects Model and Tukey's multiple comparisons testing. Mean differences in the time required for tumors to increase 2-fold or 5-fold over the size at day 1 (start of treatment) were compared using one-way Analysis of Variance (ANOVA) and Tukey's multiple comparisons testing. Survival analysis was performed using the Kaplan-Meier method and outcomes with monotherapy were compared to the combination or the monotherapies were compared to each other by the log-rank test. The significance level was p ≤ 0.05.

### Case report

The patient signed a written informed consent for the scientific publication of the medical data. Due to large tumoral masses within the pelvis (**Figure 4**), a proper dose escalation using EBRT (>50 Gy EQD2) was not feasible without causing harm to neighboring organs at risk as the bladder and the bowel. Consequently, the decision to combine elective EBRT with PSMA-RLT to escalate the dose was taken based on the recommendations of the urogenital tumorboard of the Medical Center - University of Freiburg. EBRT was delivered from 07/2015-08/2015 with intensity-modulated radiotherapy (IMRT) including cone-beam positioning verification. The target volumes were delineated according to the Radiation Therapy Oncology Group (RTOG) criteria. Radiotherapy was administered five times per week in 1.8 Gy fractions to the prostate (total dose: 54 Gy) and pelvic lymphatics (total dose: 45 Gy). After radiotherapy, three cycles of [^177^Lu]Lu-PSMA-617 therapy were then administered from 11/2015-05/2016 (3.4-6.0 GBq per cycle, 14.9 GBq in total). After completion of the last PSMA-RLT cycle, follow-up examinations were performed every three months including regular blood tests and renal scanning with technetium-99m-mercaptoacetyltriglycine ([^99m^Tc]Tc-MAG3).

## Results

### PSMA protein and mRNA levels are upregulated after irradiation

**Figure 1:**
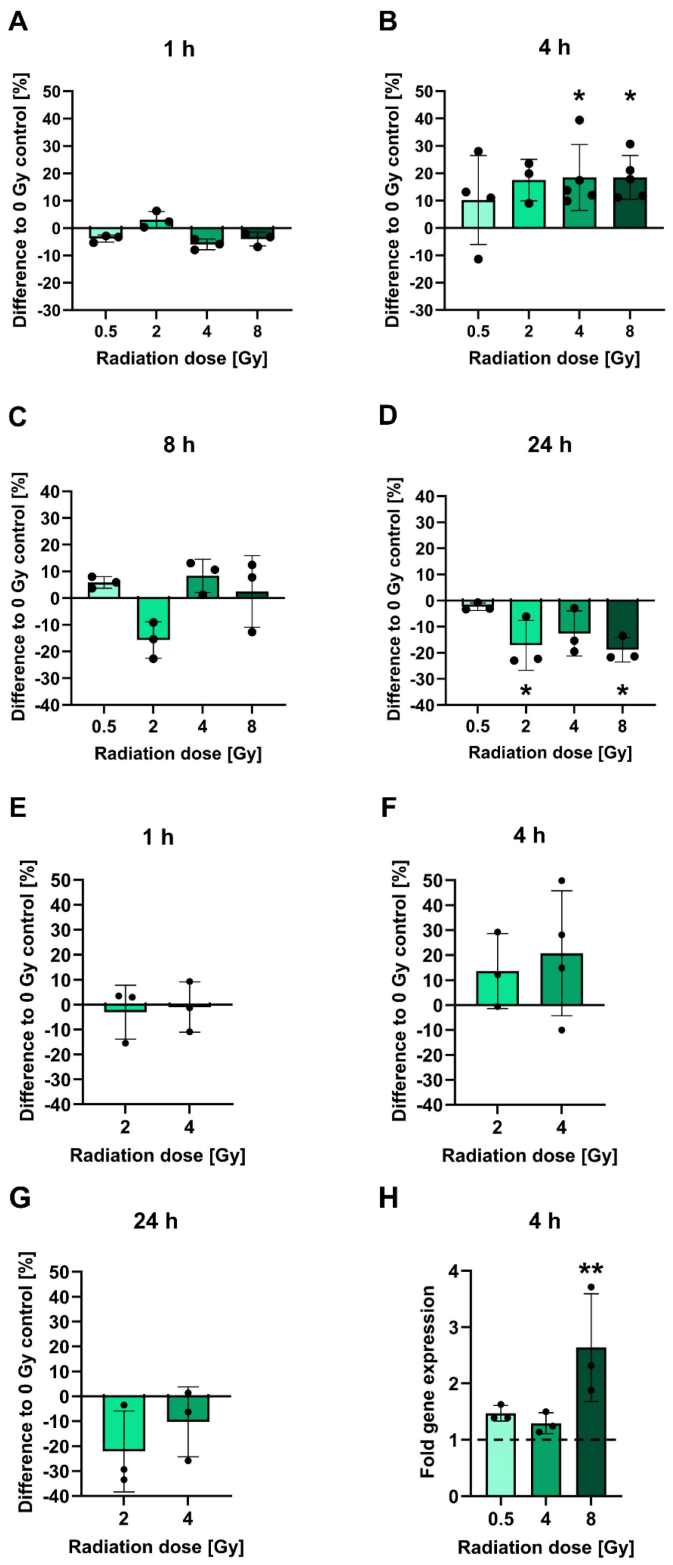
Analysis of PSMA protein and gene expression after irradiation. **A-D:** PSMA expression levels on the surface of LNCaP cells measured using flow cytometry at 1 h (**A**), 4 h (**B**), 8 h (**C**) or 24 h (**D**) post-irradiation with 0.5-8 Gy. **E-G:** Confirmation of changes in PSMA protein expression in 2 or 4 Gy irradiated LNCaP cells. PSMA protein levels on the cell surface were measured by OCW at 1 h (**E**), 4 h (**F**) and 24 h (**G**) post-irradiation. PSMA-derived fluorescence signals were normalized to fluorescence of a non-specific cell stain. **H:** Analysis of *FOLH1* mRNA induction in LNCaP cells by RT-qPCR at 4 h post-irradiation with 0.5-8 Gy. *FOLH1* expression was normalized to *GAPDH*. The dashed line marks the *FOLH1* expression level in non-irradiated control cells. All data is presented as mean ± SD. Asterisks mark significant differences to non-irradiated control cells: Kruskal-Wallis test and Dunn's multiple comparisons test: * p < 0.05, ** p < 0.01. Abbreviations: FOLH1: folate hydrolase 1; GAPDH: glyceraldehyde-3-phosphate dehydrogenase; OCW: On-Cell Western Assay; PSMA: prostate-specific membrane antigen; RT-qPCR: reverse transcription quantitative polymerase chain reaction.

To investigate the influence of irradiation on PSMA protein levels, LNCaP cells cultured *in vitro* were subjected to cell surface staining of PSMA at 1-24 h following irradiation with 0.5-8 Gy and analyzed by flow cytometry (**Figures 1A-D**). At an early time point of 1 h post-irradiation, no significant alterations in PSMA levels relative to non-irradiated cells were evident. The PSMA levels then increased by 4 h post-irradiation in a manner that did not seem to be dose-dependent: Cells irradiated with 2 Gy displayed an upregulation of surface PSMA levels by 17.5%. After 4 or 8 Gy irradiation, the levels were significantly upregulated by 18.5% for both doses (p = 0.0500 and p = 0.0244, respectively). Later than 4 h post-irradiation, PSMA expression was steadily declining. By 24 h, irradiated cells had up to 18.9% (p = 0.0412) lower surface PSMA levels as compared to non-irradiated cells. Downregulated levels of PSMA persisted until 48 h following irradiation (**Table S2**). All measured changes in PSMA levels for each experiment and p-values for comparisons of the irradiated to non-irradiated conditions are listed in **Table S2**. A similar pattern of radiation-induced changes in PSMA expression was confirmed in OCW assays also detecting PSMA on the cell membrane. However, the experiments were performed on fixed, non-viable cells without prior detachment by trypsinization, and using a different primary antibody (**Figure 1E-G**). In comparison to the control cells, small average changes in PSMA expression levels and overall variable effects were found at 1 h, an upregulation by 13.6-20.7% was measured at 4 h and a downregulation by up to 22.1% was evident at 24 h post-irradiation (for exact values, see **Table S2**). The specificities of the PSMA-reactive antibodies were verified using an IgG1 isotype control (flow cytometry) (**Figure S3A-C**) or the PSMA-negative PC-3 cells (OCW) (**Figure S3D**). Additionally, only the secondary antibody used in OCW assays was added to the cells and unspecific signals were not detected (**Figure S3D**).

To assess whether the observed surface PSMA upregulation after irradiation is due to increased protein translocation to the membrane or *de novo* protein synthesis, *FOLH1* mRNA levels in LNCaP cells were analyzed by RT-qPCR at 4 h following radiation exposure (**Figure 1H**). An induction of mRNA was already observed with a dose of 0.5 Gy evident from a 1.5-fold increase over untreated controls. With 4 Gy and 8 Gy irradiation, there was a 1.3-fold and 2.6-fold (p = 0.0061) increase in mRNA levels, respectively (for all fold change values and p-values, see **Table S3**).

### Combining EBRT and PSMA-RLT significantly improves outcomes *in vivo*

**Figure 2:**
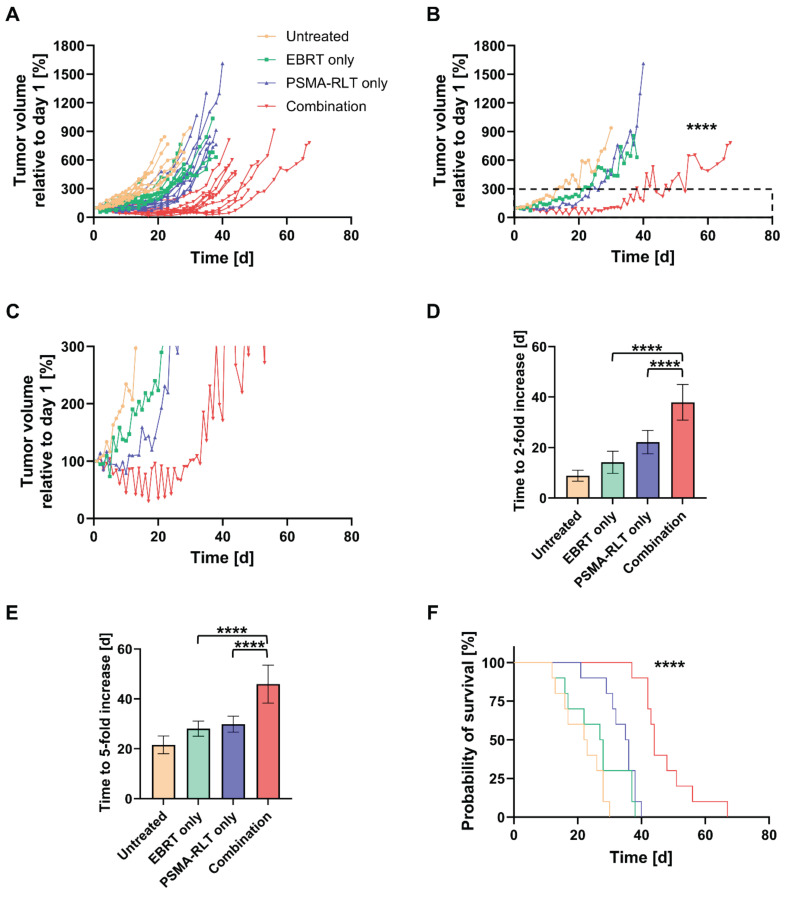
*In vivo* therapy study of the combination of 2 Gy EBRT and 40 MBq [^177^Lu]Lu-PSMA-617 (PSMA-RLT). **A:** Development of the tumor growth of all individual animals. In the combination group, PSMA-RLT was administered 4 h after EBRT on day 1. **B:** Mean tumor growth per group. Insert marks area enlarged in **C** that shows the early shrinkage of tumors treated with the combination. Time to 2-fold (**D**) or 5-fold (**E**) increase in tumor size (mean ± SD) relative to day 1. **F:** Survival according to Kaplan-Meier. Asterisks indicate significant differences for the combination therapy vs. each monotherapy: The course of tumor growth (**B**) was compared using the Mixed-Effects Model and Tukey's multiple comparisons test, and differences in the time to a 2-fold (**D**) or 5-fold increase (**E**) in tumor size were analyzed by one-way ANOVA and Tukey's multiple comparisons testing. Differences in survival were assessed using the log-rank test; **** p < 0.0001. n = 10 per group. Abbreviations: ANOVA: analysis of variance; EBRT: external beam radiotherapy; PSMA-RLT: prostate-specific membrane antigen-targeted radioligand therapy.

An *in vivo* therapy study was conducted to evaluate the efficacy of combined EBRT and PSMA-RLT for PC. Based on the *in vitro* finding of increased surface PSMA levels on PC cells after irradiation, the therapy study was designed to administer EBRT first followed by PSMA-RLT with a delay of 4 h. The combination of 2 Gy EBRT and 40 MBq [^177^Lu]Lu-PSMA-617 significantly delayed LNCaP tumor growth as compared to each monotherapy (both p < 0.0001) (**Figures 2A-B**, **Table S6**). Additionally, all tumors in the combination group showed an initial decrease in size and the mean tumor size remained smaller than prior to treatment for 30 days (**Figure 2C**). The courses of the mean tumor growth of the two monotherapies were not found to be significantly different (**Table S6**). The tumor growth outcomes are displayed for all mice separated by group in **Figure S4** (for the mean values, see **Table S4**). When compared to monotherapy with EBRT or PSMA-RLT, the tumor doubling time was significantly extended 2.7-fold or 1.7-fold with combination therapy, respectively (**Figure 2D**, **Table S6**; both p < 0.0001). A statistical comparison of the tumor doubling time between EBRT alone and PSMA-RLT alone showed a significant difference (p = 0.0058) with RLT achieving longer doubling times. Later on, the monotherapies were equally effective as evidenced by the time required for a 5-fold enlargement of the tumor (**Figure 2E**, **Table S6**). On the other hand, the combined treatment was still superior over the monotherapies significantly prolonging the time to 5-fold enlargement (both p < 0.0001).

The survival of the mice was also significantly improved with the combination therapy as compared to each monotherapy (**Figure 2F**, both p < 0.0001). Median survival times amounted to 44 d, 35.5 d, 27.5 d and 22.5 d in the combination, PSMA-RLT only, EBRT only and untreated groups, respectively (for the survival time of each animal, see **Table S5**). Relative to no treatment, this means that EBRT extended survival by 5 d, PSMA-RLT by 13 d and the combination almost doubled the survival time (+21.5 d). Despite the longer median survival time observed with PSMA-RLT as compared to EBRT alone, the difference was not significant (**Table S6**).

### Tumor-absorbed doses from RLT are somewhat enhanced with prior EBRT

**Figure 3:**
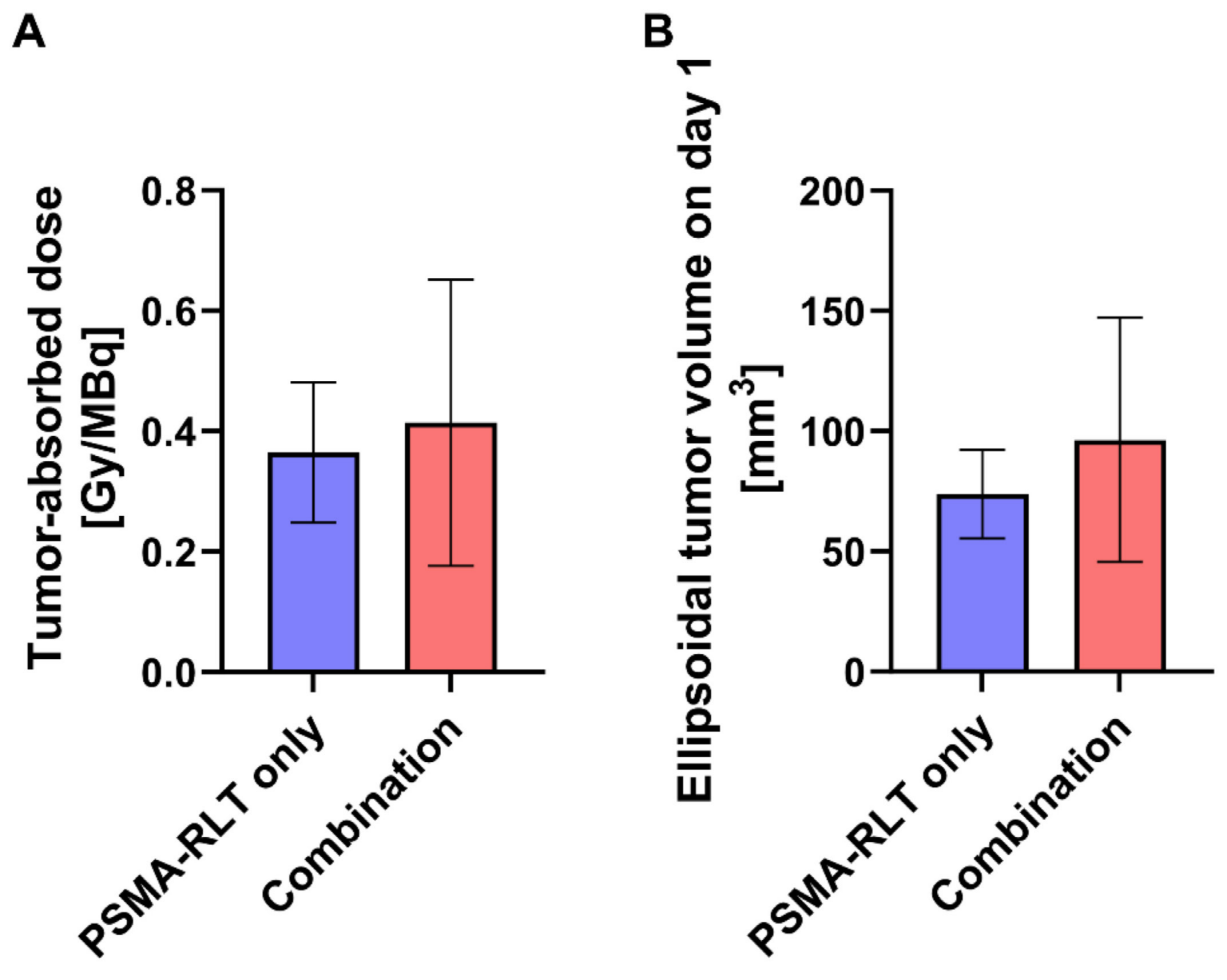
Calculated tumor-absorbed doses by [^177^Lu]Lu-PSMA-617 and ellipsoidal tumor volumes by treatment group. **A:** Tumor-absorbed doses were calculated according to the MIRD formalism following the determination of time-activity curves using a gamma probe. Cumulative doses are shown for the time from injection of activity until day 21 which corresponds to the shortest registered survival time. The difference in tumor-absorbed doses between the combination treatment and PSMA-RLT only was not significant (t test with Welch's correction, p = 0.5652). **B:** Tumor volumes were calculated considering the tumors as ellipsoids. The difference in tumor volumes between the combination treatment and PSMA-RLT only groups was not significant (t test with Welch's correction, p = 0.2138). The graphs show the mean ± SD. n = 10 per group. Abbreviations: MIRD: Medical Internal Radiation Dosimetry; PSMA-RLT: prostate-specific membrane antigen-targeted radioligand therapy.

To assess whether prior EBRT enhanced [^177^Lu]Lu-PSMA-617 uptake in the combination group, tumor-absorbed doses for mice receiving RLT were determined. As differences in the survival time of the animals would affect the calculated cumulative tumor-absorbed dose, the shortest registered survival time (21 d) served as a time limit for the dose calculations. The achieved doses in the PSMA-RLT and combination groups varied considerably (**Figure 3A**) and amounted to 0.36 ± 0.12 Gy/MBq and 0.41 ± 0.24 Gy/MBq, respectively. However, the average group difference of 13.9% did not attain statistical significance. Interestingly, the extent of enhancement of the tumor-absorbed dose in the combination group fits to the comparably small upregulation of PSMA protein levels in response to 2 Gy irradiation detected *in vitro* (+17.5%; **Figure 1B**). Tumor-absorbed doses for each animal applying different time limits for determination of cumulated doses are listed in **Table S7**. The calculated ellipsoidal tumor volumes were 37% higher in the combination group than in the PSMA-RLT group (**Figure 3B**) which might affect the comparability of the results. However, this difference was caused by 3/10 animals in the combination group and was not statistically significant.

### First patient responds with lasting remission

**Figure 4:**
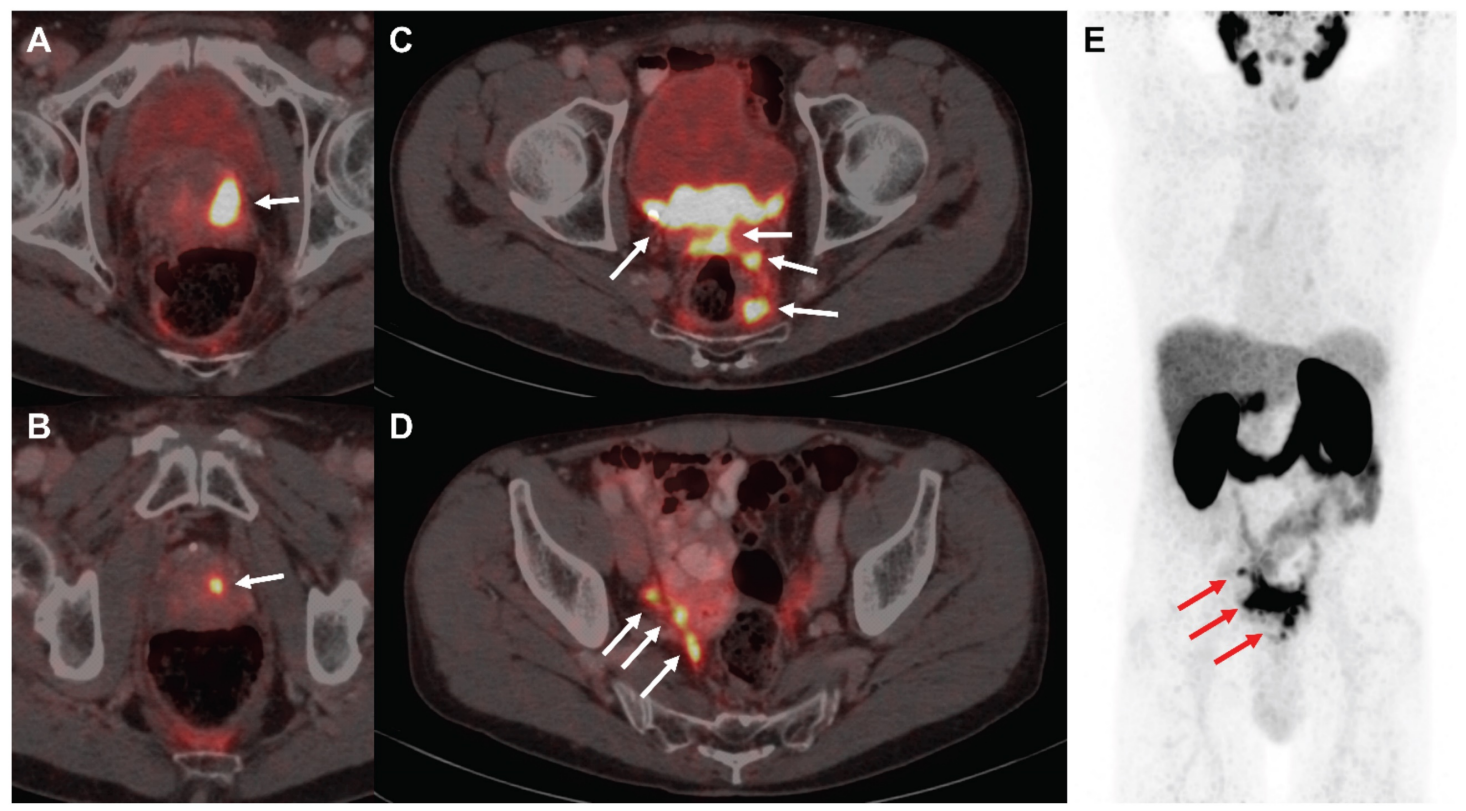
[^68^Ga]Ga-PSMA-11 PET/CT images of the exemplified patient undergoing EBRT followed by PSMA-RLT for locally advanced PC. The patient suffered from multifocal prostate carcinoma (**A, B**), multiple pelvic nodular tumor lesions with bladder infiltration (**C**) and lymph node and possible peritoneal metastases in the right internal iliac artery region (**D**) in PSMA-PET/CT imaging, as indicated by white arrows, respectively. Maximum intensity projection (**E**) demonstrates absence of organ metastasis, tumor lesions from **A-D** are depicted using red arrows. Abbreviations: EBRT: external beam radiotherapy; PSMA-PET/CT: prostate-specific membrane antigen-positron emission tomography/ computed tomography; PSMA-RLT: prostate-specific membrane antigen-targeted radioligand therapy.

A 74-year old patient (**Figure 4**) was newly diagnosed with locally advanced prostate adenocarcinoma Gleason 9 cT3b cN1 cM0 (International Society of Urological Pathology (ISUP) grade group 4), with positive biopsy probes in 8/8 samples and an initial prostate-specific antigen (iPSA) level of 4.69 ng/mL. The patient was in good health (Eastern Cooperative Oncology Group (ECOG) grade 0) and the initial examination revealed urinary retention grade III and suspect rectal infiltration. Androgen deprivation therapy (leuprorelin) was administered from 06/2015-05/2018. As the patient had elevated liver enzymes after flutamide and bicalutamide treatment, no other antiandrogens were given. Furthermore, the patient refused chemotherapy. Due to the extended tumoral masses in the pelvis a proper dose coverage was not possible by using EBRT only. From 07/2015-08/2015, the patient received IMRT to the prostate (54 Gy) and pelvic lymphatics (45 Gy) in 25 fractions. From 11/2015, he received three cycles of [^177^Lu]Lu-PSMA-617 therapy until 05/2016 (3.4-6.0 GBq per cycle). In the first cycle, 3.4 GBq were administered. Two months later, the patient received 6.0 GBq in the second cycle and after another 3.5 months 5.5 GBq in the third cycle. Overall, the treatment was well tolerated with acute and chronic genitourinary/gastrointestinal toxicities of 2/1 and 1/0 according to the Common Terminology Criteria for Adverse Events version 4.1 (CTCAEv4.1), respectively. Additionally, no hematological or renal toxicities were observed after PSMA-RLT. However, the patient reported a chronic CTCAEv4.1 grade 1 salivary gland dysfunction without dietary alterations. The patient exhibited a complete biochemical remission that lasted for five years following the last PSMA-RLT cycle. In 04/2021, the patient presented with elevated PSA of 0.48 ng/mL and a [^68^Ga]Ga-PSMA-11 positron emission tomography/computed tomography (PET/CT) was conducted which revealed progression by showing a new rib and lymph node metastasis. Both metastases occurred outside of the irradiation field.

## Discussion

One advantage of combination therapies in cancer treatment is the possibility of escalating anti-tumor effects while avoiding excessive toxicity to healthy tissue. The aim of this study was to investigate whether combining EBRT and PSMA-RLT with [^177^Lu]Lu-PSMA-617 can enhance the therapeutic strength in PC treatment. Besides an augmentation of the radiation dose by combining two types of radiotherapy, there also might be the potential of influencing the PSMA expression on the cell surface of PC cells by EBRT. An upregulation of PSMA could improve the success of subsequent RLT by increasing the uptake of PSMA-targeting radiopharmaceuticals. Working on this hypothesis, the present *in vitro* study discovered an upregulation of PSMA gene and protein expression levels on LNCaP cells 4 h after exposure to external radiation. The effect occurred after a single fraction of irradiation at clinically relevant doses. Furthermore, the findings indicate that the kinetics of PSMA surface expression is rather fast, switching from an increase to decrease of membrane localization relative to non-irradiated cells within only 24 h. Such a transient nature of upregulation of PSMA protein levels upon irradiation might pose a challenge for clinical translation of the combination therapy.

Other groups have also investigated the effects of external ionizing radiation on the protein and gene expression of PSMA **22,23**. A 2-fold upregulation of PSMA protein levels in LNCaP cells was reported for the time point of 24 h after fractionated irradiation (six fractions of 2 Gy delivered during two weeks) **22**. This significantly longer time frame might facilitate the clinical translation of combined EBRT and RLT. More in line with the results of this study showing that PSMA is downregulated by 24 h post-irradiation and the effect persists until 48 h, another group also found that PSMA expression decreased at later time points following irradiation of LNCaP cells **23**. The authors reported a decline in PSMA protein levels by a third at 48 h after 8 Gy irradiation. Such an effect might be related to a restructuring of the cell membrane as a consequence of induction of cell death since 8 Gy irradiation was reported to diminish survival of LNCaP cells by 80% 24. An initial PSMA upregulation and subsequent downregulation to levels lower than at baseline support the concept of an adaptive response of the high PSMA-expressing LNCaP cells towards radiation exposure. It can be assumed that upon initial enhancement of PSMA localization to the cell membrane shortly after irradiation, the protein is internalized. The reason for that might lie in the involvement of the glutamate carboxypeptidase PSMA in folate metabolism. The protein can cleave poly-γ-glutamated folates to their bioavailable monoglutamated form, thereby providing enzyme cofactors for nucleotide synthesis **1**, a process that is crucial for efficient DNA damage repair **25**. During the time when PSMA is upregulated on the cell membrane, it might stimulate PI3K/ Akt signaling **10**, a survival-related pathway that is known to be engaged in cancer cells in response to irradiation and which facilitates the repair of DSBs via the non-homologous endjoining pathway **11**. On the other hand, PSMA internalization at later time points might serve the purpose of transporting folate into the cell **26**.

Translating the *in vitro* findings to an *in vivo* therapy study, a single fraction of EBRT delivering a clinically relevant dose of 2 Gy followed by 40 MBq of PSMA-RLT resulted in a long-lasting stabilization of tumor growth. The differences in tumor growth and survival outcomes between the combination and each monotherapy were highly significant. Indeed, administering EBRT prior to RLT led to higher tumor-absorbed doses by [^177^Lu]Lu-PSMA-617, as it was hypothesized based on the observed PSMA upregulation at 4 h post-irradiation *in vitro*. The results should, however, be interpreted with caution since the enhancement of the tumor-absorbed dose was small (+14%) and the variability of the data was high. The dosimetry was affected by some uncertainties. The tumor volumes on the day of injection were larger in the combination group than in the PSMA-RLT only group (although not significantly different). This had an influence on the dosimetry according to the MIRD formalism since the S value is inversely related to the tumor volume. With higher volumes and lower S values the calculated tumor-absorbed dose is lower. Therefore, a more pronounced increase in the tumor-absorbed dose might have been found after combination therapy relative to RLT alone if the tumor volumes in both groups had been equal. The accuracy of dosimetry calculation was additionally affected by uncertainties in the caliper measurements of the tumor size. Regarding further improvements of the efficacy of the combination therapy, future studies might explore the possibility of achieving a complete remission of the LNCaP tumors with additional EBRT or RLT after the initial combination treatment. A suitable time point to administer additional EBRT or RLT could be at four weeks after the first treatment since that represents the time point when tumors started regrowing in the combination group. Dietrich et al. demonstrated that fractionated photon EBRT and [^90^Y]Y-Cetuximab led to a lasting tumor control in a xenograft model of head and neck squamous cell carcinoma in all tested mice **27**. Another approach could be the augmentation of RLT with alpha emitters since, due to their high LET, DNA damage might be potentiated and tumor regions with low oxygenation and acquired radiation resistance might be eradicated more efficiently **28,29**. Furthermore, as PC lacks responsiveness to immunotherapy due to its immunosuppressive tumor microenvironment **30** and low tumor mutational burden **31**, it could be explored whether the combination of EBRT and PSMA-RLT might stimulate the host immunity to the tumor and improve the efficacy of immunotherapy. Combining PSMA-617 labeled with the alpha emitter actinium-225 and subsequent administration of an anti-programmed death 1 (PD-1) antibody was previously demonstrated to enhance tumor control **32**.

Clinical experience of combined elective EBRT and dose escalation with PSMA-RLT in locally advanced PC represents a promising therapeutic approach as demonstrated in an exemplified patient case. Three cycles of PSMA-RLT were initiated three months after conclusion of the EBRT sessions and the patient was on concurrent androgen deprivation therapy while receiving EBRT and PSMA-RLT and thereafter. The combinational treatment led to a long-lasting response as evident from undetectable PSA values maintained for nearly five years after PSMA-RLT. The extent of toxicity after IMRT was low and expected for standard fractionation **33** and there was only minor chronic salivary gland toxicity associated with RLT. This promising experience might pave the way for the application of combined elective, large field EBRT, PSMA-RLT and androgen deprivation therapy for local dose escalation in more patients with locally advanced or regional PC. Future studies focussing on this clinical scenario should compare the biological effects of the cumulative doses of the different radiation types in the target region and the adjacent organs at risk. Additionally, the optimal sequencing between the treatment modalities should be identified. Our preclinical study results suggest that it might be worth exploring the outcomes of administering RLT in terms of local dose escalation a few hours after a first fraction of EBRT. Whether EBRT can alter surface PSMA expression levels in patient tumors should also be investigated. Besides locally advanced and regional PC, the combination therapy is tested in other clinical scenarios like *de novo* oligo-metastatic or oligo-recurrent PC. A reverse treatment sequence administering two cycles of [^177^Lu]Lu-PSMA-617 prior to hypofractionated stereotactic body radiotherapy for oligo-recurrent disease also showed good tolerability **8**. A similar scenario is currently being tested in the prospective LUNAR study NCT05496959.

Taken together, the combination of photon EBRT and subsequent beta-emitting PSMA-RLT in a murine PC model and an exemplified patient case with locally advanced PC showed promising disease control providing a proof-of-concept for further clinical evaluation of this therapeutic approach.

## Conclusion

The combined results of this study demonstrate a clear benefit of combining photon EBRT and PSMA-RLT with [^177^Lu]Lu-PSMA-617 for tumor control and survival in a preclinical PC model. The *in vitro* observation of radiation-induced upregulation of PSMA protein expression levels on the cell surface suggests an adaptive response of the cancer cells towards external photon irradiation. Indeed, prior EBRT seemed to enhance the *in vivo* tumor-absorbed doses after [^177^Lu]Lu-PSMA-617 treatment with, however, high variability in the data. Further research might investigate whether similar effects on PSMA expression levels and absorbed doses by the radiopharmaceutical occur in patient tumors in response to EBRT. Nevertheless, an exemplified clinical case report on this combinational approach demonstrated a lasting response and good tolerability. Further clinical investigations are crucial to gain more knowledge on the safety and efficacy of this promising therapeutic concept.

## Supplementary Material

Supplementary figures and tables.

## Abbreviations

[^177^Lu]Lu-PSMA-617: lutetium-177 (^177^Lu)-labeled PSMA-617
ANOVA: analysis of variance
cDNA: complementary DNA
Ct: threshold cycle
CTCAEv4.1: Common Terminology Criteria for Adverse Events version 4.1
DSB: DNA double strand break
EBRT: external beam radiotherapy
ECOG: Eastern Cooperative Oncology Group
EQD2: equivalent dose in 2 Gy fractions
FBS: fetal bovine serum
FOLH1: folate hydrolase 1
FSC: forward scatter
GAPDH: glyceraldehyde-3-phosphate dehydrogenase
HEPES: 2-[4-(2-hydroxyethyl)piperazin-1-yl]ethanesulfonic acid
HPLC: high performance liquid chromatography
IgG: immunoglobulin G
IMRT: intensity-modulated radiotherapy
ISUP: International Society of Urological Pathology
iPSA: initial prostate-specific antigen
LET: linear energy transfer
mCRPC: metastatic castration-resistant prostate cancer
MIRD: Medical Internal Radiation Dosimetry
NCBI: National Center for Biotechnology Information
OCW: On-Cell Western Assay
PBS: phosphate buffered saline
PC: prostate cancer
PET/CT: positron emission tomography/ computed tomography
PI: propidium iodide
PI3K: phosphatidylinositol-3-kinase
PSA: prostate-specific antigen
PSMA: prostate-specific membrane antigen
PSMA-RLT: prostate-specific membrane antigen-targeted radioligand therapy
RLT: radioligand therapy
RPMI: Roswell Park Memorial Institute
RT: room temperature
RT-qPCR: reverse transcription quantitative polymerase chain reaction
RTOG: Radiation Therapy Oncology Group
SD: standard deviation
SSB: DNA single strand break
SSC: side scatter
TLC: thin-layer chromatography
TIA: time-integrated activity
