# Supplementary material for: Exploring the role of combined external beam radiotherapy and targeted radioligand therapy with [177Lu]Lu-PSMA-617 for prostate cancer - from bench to bedside
Source: Theranostics. 2024 Apr 8;14(6):2560–72. doi: 10.7150/thno.93249 (PMC11024848; doi:10.7150/thno.93249)
Supplement: Supplementary file 1 — Supplementary figures and tables. [file thnov14p2560s1.pdf]

Supplementary Material

Exploring the role of combined external beam radiotherapy and targeted radioligand therapy with [^{177}Lu]Lu-PSMA-617 for prostate cancer – from bench to bedside

Daria Arbuznikova[1,2,3], Aikaterini Klotsotyra[1], Lisa Uhlmann[1,2], Lisa-Charlotte Domogalla[1,2], Nils Steinacker[1,2], Michael Mix[1], Gabriele Niedermann[3,4], Simon Spohn[3,4,5], Martin T. Freitag[1], Anca L. Grosu[3], Philipp T. Meyer[1], Christian Gratzke[6], Matthias Eder[1,2], Constantinos Zamboglou[3,7,#], Ann-Christin Eder[1,2,#]

Supplementary Figures 2

Figure S1: Gating strategy in the flow cytometry experiments. 2

Figure S2: Mean tumor size with SD on the day of treatment (= day 1) for each group. 3

Figure S3: Negative controls for the analysis of PSMA protein levels. 4

Figure S4: Time courses of individual tumor growth in each treatment group. 5

Supplementary Tables 6

Table S1: Primer sequences and properties. 6

Table S2: Changes in PSMA protein levels after irradiation (IR) measured by flow cytometry or OCW (mean values from all experiments (n = 3-5) and p-values). 7

Table S3: Changes in PSMA fold gene expression after irradiation (IR) measured by RT-qPCR (mean values from all experiments (n = 3) and p-values). 9

Table S4: Mean tumor volumes [%] by day normalized to the day of treatment = day 1. 10

Table S5: Survival time [d] for each animal within a treatment group. 13

Table S6: p-values for comparisons of differences regarding tumor control and survival outcomes between treatment groups in the *in vivo* therapy study. 14

Table S7: Tumor-absorbed doses [Gy/MBq] for each animal that received [^{177}Lu]Lu-PSMA-617. 15

Supplementary Figures

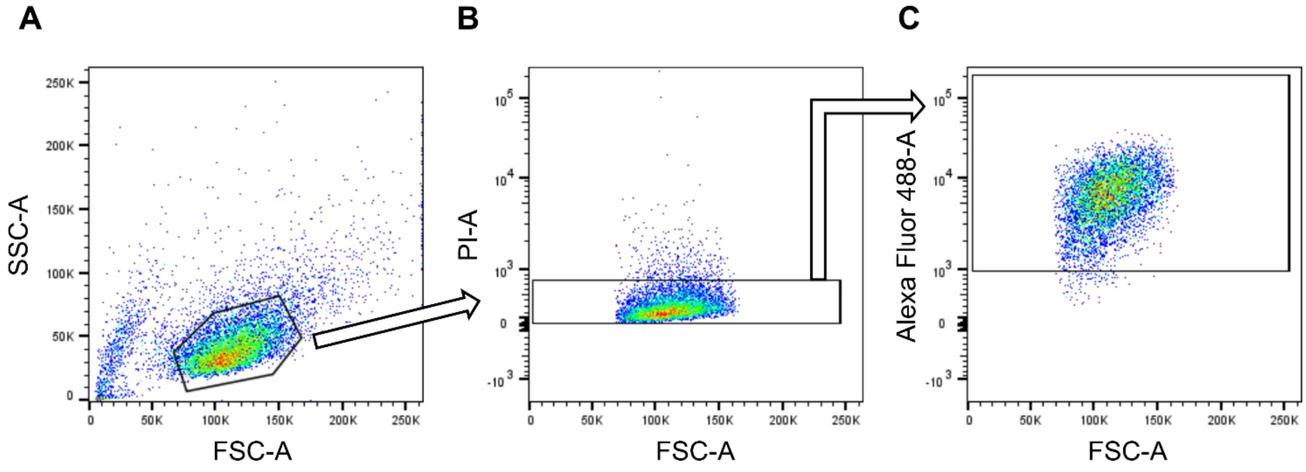

Figure S1: Gating strategy in the flow cytometry experiments. The main cell population was selected (**A**), dead cells were excluded by PI-staining (**B**) and the median fluorescence intensity in the Alexa Fluor™ 488 channel was analyzed (**C**).

Abbreviations: Alexa Fluor 488-A: Alexa Fluor 488 – pulse area; FSC: forward scatter – pulse area; PI: propidium iodide; SSC: side scatter – pulse area.

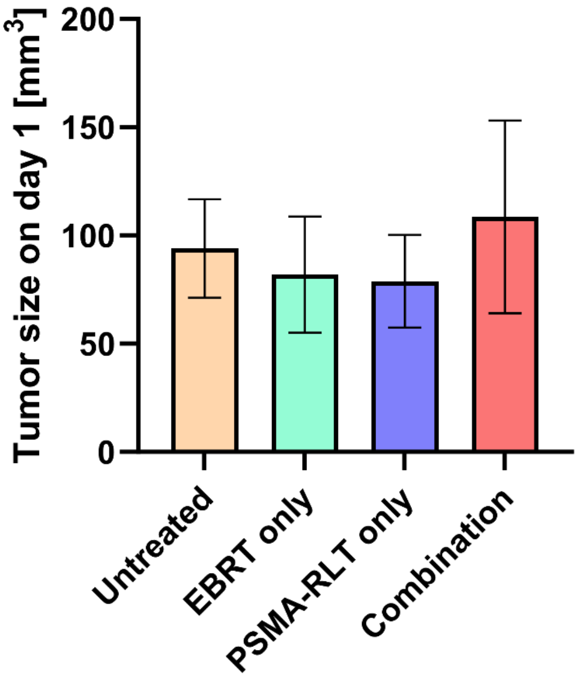

Figure S2: Mean tumor size with SD on the day of treatment (= day 1) for each group. There were no significant differences between groups (one-way ANOVA, $p = 0.1410$).

Abbreviations: ANOVA: analysis of variance; EBRT: external beam radiotherapy; PSMA-RLT: PSMA-targeted radioligand therapy; SD: standard deviation.

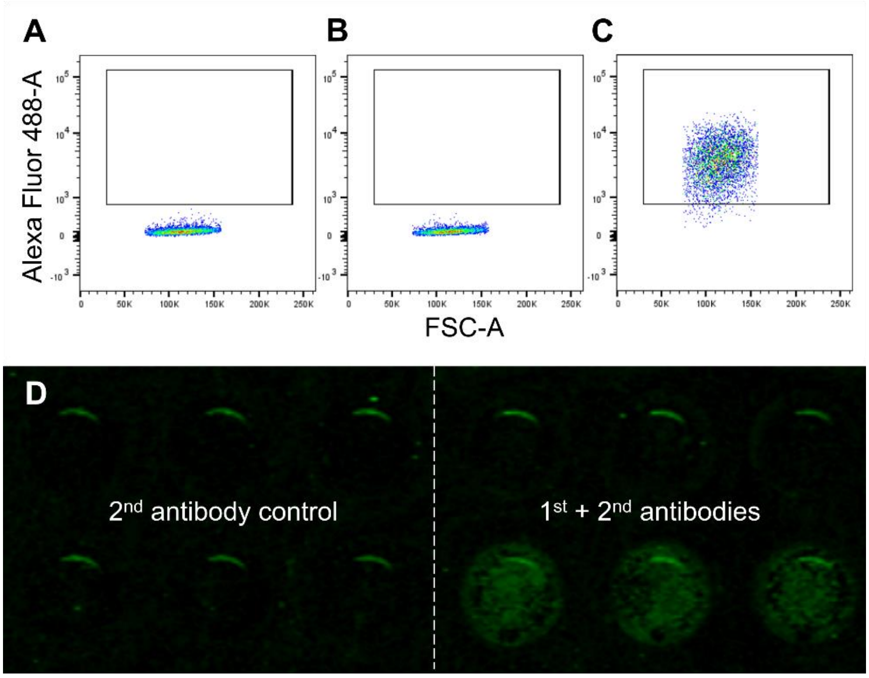

Figure S3: Negative controls for the analysis of PSMA protein levels. **A-C:** Controls for flow cytometry. Signal intensities in the Alexa Fluor™ 488 channel (Alexa Fluor 488-A) were measured for unstained cells (**A**), IgG1 isotype control-stained cells (**B**), and cells stained with the PSMA-reactive antibody (**C**). **D:** Negative controls for OCW included LNCaP cells stained only with the secondary antibody (bottom row, left) and PC-3 cells (top row) with both the primary and secondary antibodies. No signals were detected as compared to LNCaP cells stained with both antibodies (bottom row, right). Abbreviations: Alexa Fluor 488-A: Alexa Fluor 488 – pulse area; FSC: forward scatter – pulse area; IgG1: immunoglobulin G1; OCW: On-Cell Western Assay; PSMA: prostate-specific membrane antigen.

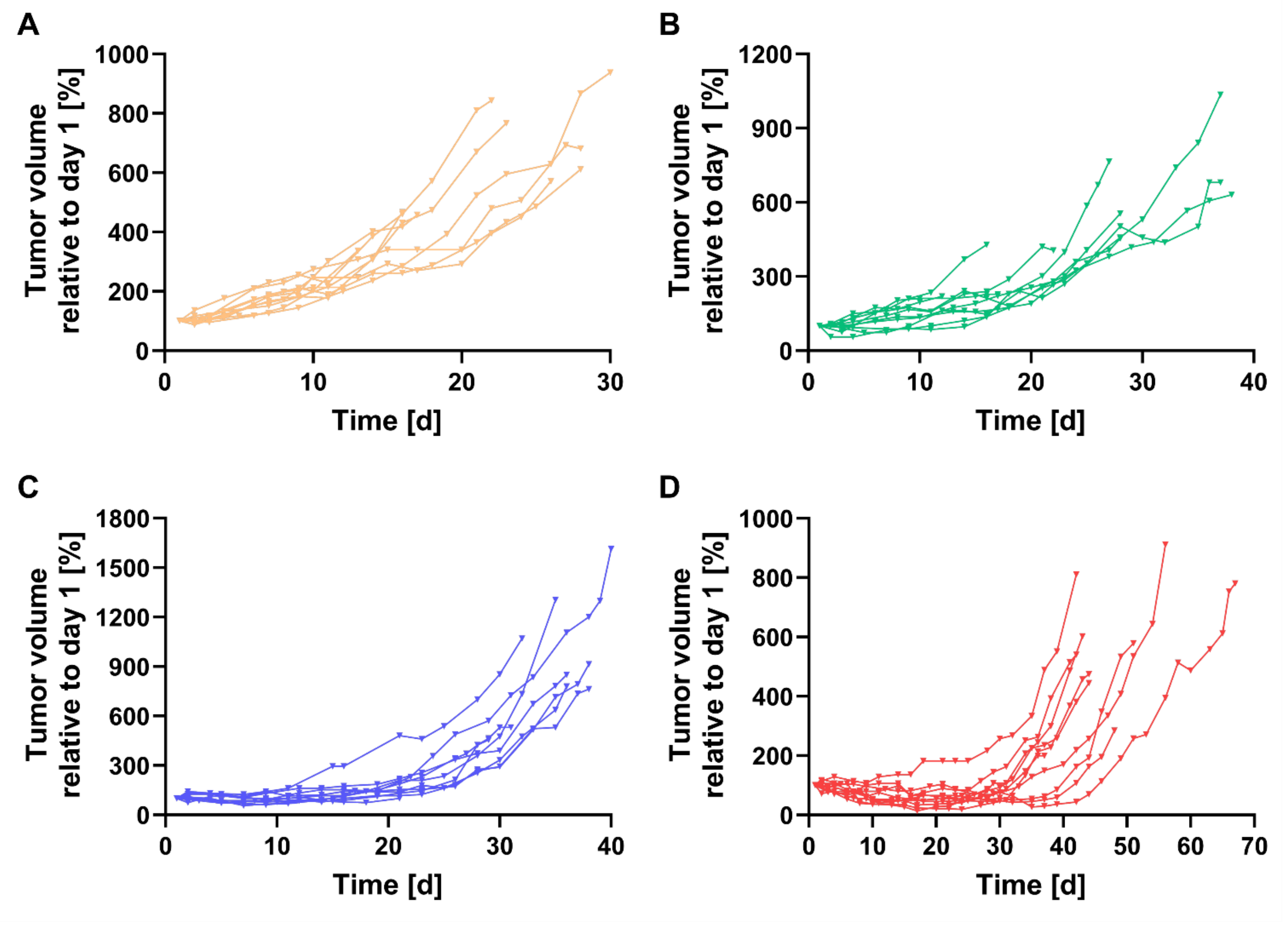

Figure S4: Time courses of individual tumor growth in each treatment group. All animals from the untreated (**A**), EBRT (**B**), PSMA-RLT (**C**) and combination groups (**D**) are displayed. Please note the differences in scaling of the x- and y-axes.

Abbreviations: EBRT: external beam radiotherapy; PSMA-RLT: PSMA-targeted radioligand therapy.

Supplementary Tables

Table S1: Primer sequences and properties.

**Target**	**Sequence**	**GC [%]**	**T_m [°C]**	**Amplicon size [bp]**	**Spanning two exons?**
FOLH1	Forward: AGTGCTCCCTTTTGATTGTCG	48	57.9	281	Yes
	Reverse: GCCTGTCTGGTAACCCTAATGG	55	62.1		
GAPDH	Forward: ACAACTTTGGTATCGTGGAAGG	45	58.4	101	No
	Reverse: GCCATCACGCCACAGTTTC	58	58.8		

Abbreviations: bp: base pairs; FOLH1: folate hydrolase 1; GAPDH: glyceraldehyde-3-phosphate dehydrogenase; GC: guanine/cytosine content; T_m: melting temperature.

Table S2: Changes in PSMA protein levels after irradiation (IR) measured by flow cytometry or OCW (mean values from all experiments (n = 3-5) and p-values).

			**Flow cytometry**		
			Difference to 0 Gy control [%]		
Comparison	***1 h post-IR***	***4 h post-IR***	***8 h post-IR***	***24 h post-IR***	***48 h post-IR***
0 vs. 0.5 Gy	-3.22	+13.15	+5.93	-3.37	-
	-5.31	+28.06	+3.69	-0.71	-
	-2.86	-11.34	+7.98	-3.10	-
	-	+11.05	-	-	-
0 vs. 2 Gy	+0.35	+9.06	-22.67	-22.99	-21.22
	+6.31	+19.91	-15.34	-22.28	+5.79
	+2.37	+23.60	-9.12	-6.11	-19.67
0 vs. 4 Gy	-7.99	+13.75	+13.09	-2.90	-
	-5.81	+12.00	+10.60	-19.49	-
	-4.00	+39.45	+1.27	-15.35	-
	-	+17.43	-	-	-
	-	+9.87	-	-	-
0 vs. 8 Gy	-6.82	+11.18	+12.42	-13.49	-
	-3.21	+11.75	+7.78	-21.71	-
	-1.93	+30.72	-12.73	-21.35	-
	-	+21.12	-	-	-
	-	+17.86	-	-	-
			p-values		
0 vs. 0.5 Gy	0.6775, ns	0.4766, ns	0.9346, ns	> 0.9999, ns	-
0 vs. 2 Gy	> 0.9999, ns	0.1137, ns	> 0.9999, ns	0.0180, *	0.2500, ns□
0 vs. 4 Gy	0.1116, ns	0.0500, *	0.3965, ns	0.2676, ns	-
0 vs. 8 Gy	0.6775, ns	0.0244, *	> 0.9999, ns	0.0412, *	-

OCW			
Difference to 0 Gy control [%]			
Comparison	***1 h post-IR***	***4 h post-IR***	***24 h post-IR***
0 vs. 2 Gy	-15.42	+29.22	-3.52
	+2.98	+12.16	-33.48
	+3.55	-0.61	-29.42
0 vs. 4 Gy	-1.28	+14.86	-6.30
	+9.33	-10.02	-25.86
	-10.84	+49.85	+1.39
		+28.05	
p-values			
0 vs. 2 Gy	> 0.9999, ns	0.9177, ns	0.0974, ns
0 vs. 4 Gy	> 0.9999, ns	0.4601, ns	0.8968, ns

Statistical analysis for each time point: Kruskal-Wallis test and Dunn’s multiple comparisons test.

□Wilcoxon signed-rank test (two-tailed).

ns = not significant, * = $p < 0.05$.

Abbreviations: IR: irradiation; OCW: On-Cell Western Assay; PSMA: prostate-specific membrane antigen.

Table S3: Changes in PSMA fold gene expression after irradiation (IR) measured by RT-qPCR (mean values from all experiments (n = 3) and p-values).

**RT-qPCR**			
Fold gene expression			
Time point	***0 vs. 0.5 Gy***	***0 vs. 4 Gy***	***0 vs. 8 Gy***
4 h post-IR	1.39	1.50	2.32
	1.39	1.14	3.71
	1.63	1.24	1.88
p-values			
4 h post-IR	0.2028, ns	0.6268, ns	0.0061, **

Statistical analysis: Kruskal-Wallis test and Dunn's multiple comparisons test.

ns = not significant, ** = $p < 0.01$.

Abbreviations: IR: irradiation; PSMA: prostate-specific membrane antigen; RT-qPCR: reverse transcription quantitative polymerase chain reaction.

Table S4: Mean tumor volumes [%] by day normalized to the day of treatment = day 1. Shown are mean values ± *SD*.

Day	Untreated	EBRT only	PSMA-RLT only	Combination
1	100.00 (*0*)	100.00 (*0*)	100.00 (*0*)	100.00 (*0*)
2	106.77 (*16.99*)	94.75 (*19.58*)	114.52 (*21.76*)	104.76 (*17.77*)
3	109.65 (*10.42*)	95.65 (*16.31*)	85.71	83.83 (*6.83*)
4	133.91 (*26.20*)	109.36 (*36.61*)	117.15 (*13.77*)	95.65 (*22.75*)
5	116.67	73.33	95.27 (*24.51*)	104.00
6	163.18 (*38.31*)	141.48 (*26.39*)		77.04 (*22.41*)
7	174.27 (*35.26*)	118.55 (*41.58*)	93.93 (*21.75*)	84.18 (*21.07*)
8	186.21 (*35.50*)	158.36 (*34.71*)	85.71	60.19 (*20.99*)
9	195.51 (*37.29*)	138.19 (*52.59*)	99.20 (*31.00*)	83.23 (*21.42*)
10	234.30 (*32.35*)	169.46 (*40.59*)	79.12	44.44 (*9.28*)
11	222.60 (*48.21*)	147.45 (*59.02*)	110.93 (*38.39*)	85.80 (*33.45*)
12	206.88 (*9.73*)	190.35 (*40.74*)	109.93 (*31.41*)	86.67
13	296.87 (*46.29*)	181.22 (*27.55*)		41.77 (*8.00*)
14	316.58 (*66.01*)	203.56 (*99.87*)	111.24 (*32.17*)	87.71 (*32.71*)
15	316.67 (*33.00*)	189.33 (*31.19*)	159.07 (*118.40*)	39.26 (*10.53*)
16	387.25 (*90.00*)	218.63 (*102.58*)	139.70 (*66.13*)	76.64 (*30.89*)
17	356.27 (*94.69*)	205.89 (*33.47*)	144.23	29.93 (*14.96*)
18	444.54 (*144.16*)	217.88 (*53.57*)	119.95 (*39.02*)	86.77 (*54.41*)
19	392.86	240.00	141.68 (26.29)	96.00
20	315.96 (*34.00*)	223.19 (*45.65*)		34.77 (*14.10*)
21	592.23 (*191.27*)	289.73 (*79.52*)	192.57 (*115.37*)	91.18 (*49.29*)
22	573.19 (*238.06*)	317.26 (*76.28*)	230.77	36.76 (*14.59*)
23	598.61 (*166.68*)	313.73 (*58.18*)	219.64 (*107.05*)	86.23 (*49.78*)
24	478.33 (*40.07*)	342.01 (*24.70*)	356.04	42.71 (*18.82*)

25	486.11	448.83 (*122.24*)	311.91 (*198.86*)	86.99 (*54.74*)
26	599.86 (40.60)	528.92 (199.73)	288.65 (123.17)	68.57
27	693.33	516.73 (*214.80*)	371.43	66.70 (*34.21*)
28	719.58 (*132.08*)	492.20 (*47.11*)	384.36 (*144.63*)	90.26 (*64.25*)
29		418.18	496.90 (*64.52*)	90.69 (*44.87*)
30	937.50	493.96 (*52.06*)	451.61 (*198.95*)	101.09 (*78.01*)
31		439.09	627.74 (*137.94*)	101.98 (*47.22*)
32		437.14	759.46 (*298.79*)	109.10 (*93.77*)
33		740.38	636.53 (*150.30*)	95.33
34		566.29		184.89 (*54.49*)
35		670.71 (*239.41*)	793.43 (*301.00*)	135.71 (*121.72*)
36		643.81 (*52.53*)	911.28 (*172.07*)	230.30 (*28.83*)
37		857.98 (*250.35*)	764.05 (*39.66*)	173.76 (*159.34*)
38		630.00	959.18 (*221.80*)	306.62 (*82.79*)
39			1298.08	198.05 (*216.16*)
40			1613.32	170.67
41				456.11 (*77.87*)
42				323.38 (*273.46*)
43				529.58 (*102.95*)
44				266.88 (*161.25*)
46				219.34 (*118.94*)
47				335.24
48				284.45
49				377.51 (*173.69*)
51				457.12 (*173.89*)
53				271.49
54				643.81

56	652.82 (*365.73*)
58	514.12
60	487.06
63	558.82
65	611.76
66	752.58
67	780.45

Abbreviations: EBRT: external beam radiotherapy; PSMA-RLT: prostate-specific membrane antigen-targeted radioligand therapy; SD: standard deviation.

Table S5: Survival time [d] for each animal within a treatment group.

**Animal**	**Untreated**	**EBRT only**	**PSMA-RLT only**	**Combination**
1	12	38	21	44
2	28	28	36	37
3	26	12	35	42
4	17	37	40	43
5	30	27	38	51
6	16	28	38	48
7	28	22	29	67
8	22	37	31	42
9	23	16	36	44
10	13	17	32	56
**Mean**	21.5	26.2	33.6	47.4
**SD**	6.6	9.4	5.6	8.7

Abbreviations: EBRT: external beam radiotherapy; PSMA-RLT: prostate-specific membrane antigen-targeted radioligand therapy; SD: standard deviation.

Table S6: p-values for comparisons of differences regarding tumor control and survival outcomes between treatment groups in the *in vivo* therapy study.

Tumor control			
**Parameter**	**EBRT only vs. PSMA-RLT only**	**EBRT only vs. Combination**	**PSMA-RLT only vs. Combination**
Mean tumor growth	0.9672, ns	< 0.0001, ****	< 0.0001, ****
Time to 2-fold tumor increase	0.0058, **	< 0.0001, ****	< 0.0001, ****
Time to 5-fold tumor increase	0.9047, ns	< 0.0001, ****	< 0.0001, ****
**Survival**			
Survival curve	0.1239, ns	< 0.0001, ****	< 0.0001, ****

Statistical analysis: Mixed-Effects Model and Tukey’s multiple comparisons testing (tumor growth); one-way ANOVA and Tukey’s multiple comparisons testing (time to 2-fold or 5-fold increase); Kaplan-Meier survival analysis and log-rank test (survival).

ns = not significant, ** = $p < 0.01$, **** = $p < 0.0001$.

Abbreviations: ANOVA: analysis of variance; EBRT: external beam radiotherapy; PSMA-RLT: prostate-specific membrane antigen-targeted radioligand therapy.

Table S7: Tumor-absorbed doses [Gy/MBq] for each animal that received [^{177}Lu]Lu-PSMA-617. Doses were calculated for different time periods: Day of injection until day 21 (t = 0-21); day of injection until infinity (t = 0-infinity); day of injection until the last measurement (t = 0-last data point).

**PSMA-RLT only**			
**Animal**	**t = 0-21**	**t = 0-infinity**	**t = 0-last data point**
**1**	0.59	0.68	0.58
**2**	0.42	0.46	0.40
**3**	0.46	0.49	0.45
**4**	0.22	0.22	0.20
**5**	0.38	0.40	0.33
**6**	0.43	0.43	0.39
**7**	0.25	0.26	0.23
**8**	0.38	0.39	0.32
**9**	0.27	0.28	0.24
**10**	0.25	0.25	0.24
**Mean**	**0.36**	**0.39**	**0.34**
**SD**	**0.12**	**0.14**	**0.12**
**Combination**			
**Animal**	**t = 0-21**	**t = 0-infinity**	**t = 0-last data point**
**1**	0.23	0.25	0.21
**2**	0.17	0.18	0.16
**3**	0.16	0.19	0.14
**4**	0.18	0.24	0.14
**5**	0.59	0.62	0.54
**6**	0.49	0.51	0.46
**7**	0.90	0.94	0.82
**8**	0.45	0.46	0.43

**9**	0.40	0.40	0.28
**10**	0.57	0.59	0.52
**Mean**	**0.41**	**0.44**	**0.37**
**SD**	**0.24**	**0.24**	**0.22**

Abbreviations: [^{177}Lu]Lu-PSMA-617: lutetium-177 (^{177}Lu)-labeled PSMA-617; PSMA-RLT: prostate-specific membrane antigen-targeted radioligand therapy; SD: standard deviation.
